# Post-Disaster Food and Nutrition from Urban Agriculture: A Self-Sufficiency Analysis of Nerima Ward, Tokyo

**DOI:** 10.3390/ijerph14070748

**Published:** 2017-07-10

**Authors:** Giles Bruno Sioen, Makiko Sekiyama, Toru Terada, Makoto Yokohari

**Affiliations:** 1 Graduate Program in Sustainability Science—Global Leadership Initiative, Graduate School of Frontier Sciences, The University of Tokyo, 277-8563 Chiba, Japan; sekiyama@k.u-tokyo.ac.jp; 2 Department of Natural Environmental Studies, Graduate School of Frontier Sciences, The University of Tokyo, 277-8563 Chiba, Japan; terada@k.u-tokyo.ac.jp (T.T.); myoko@k.u-tokyo.ac.jp (M.Y.); 3 Department of Urban Engineering, School of Engineering, The University of Tokyo, 113-8654 Tokyo, Japan

**Keywords:** urban agriculture, disaster, preparedness, vegetable, nutrition, emergency, self-sufficiency, public health

## Abstract

*Background*: Post-earthquake studies from around the world have reported that survivors relying on emergency food for prolonged periods of time experienced several dietary related health problems. The present study aimed to quantify the potential nutrient production of urban agricultural vegetables and the resulting nutritional self-sufficiency throughout the year for mitigating post-disaster situations. *Methods*: We estimated the vegetable production of urban agriculture throughout the year. Two methods were developed to capture the production from professional and hobby farms: Method I utilized secondary governmental data on agricultural production from professional farms, and Method II was based on a supplementary spatial analysis to estimate the production from hobby farms. Next, the weight of produced vegetables [t] was converted into nutrients [kg]. Furthermore, the self-sufficiency by nutrient and time of year was estimated by incorporating the reference consumption of vegetables [kg], recommended dietary allowance of nutrients per capita [mg], and population statistics. The research was conducted in Nerima, the second most populous ward of Tokyo’s 23 special wards. Self-sufficiency rates were calculated with the registered residents. *Results*: The estimated total vegetable production of 5660 tons was equivalent to a weight-based self-sufficiency rate of 6.18%. The average nutritional self-sufficiencies of Methods I and II were 2.48% and 0.38%, respectively, resulting in an aggregated average of 2.86%. Fluctuations throughout the year were observed according to the harvest seasons of the available crops. Vitamin K (6.15%) had the highest self-sufficiency of selected nutrients, while calcium had the lowest (0.96%). *Conclusions*: This study suggests that depending on the time of year, urban agriculture has the potential to contribute nutrients to diets during post-disaster situations as disaster preparedness food. Emergency responses should be targeted according to the time of year the disaster takes place to meet nutrient requirements in periods of low self-sufficiency and prevent gastrointestinal symptoms and cardiovascular diseases among survivors.

## 1. Introduction

Rations and emergency foods in post-disaster situations are rich in carbohydrates and focus on providing energy to survivors [1]. Such foods conventionally have a long shelf life (non-perishable). Nutrient-rich and fresh products, however, have short shelf lives [2]. Non-perishable foods have been preferred for emergency responses and meet the short-term needs of survivors. Although intended for short-term interventions lasting a few days, post-earthquake studies from around the world—including Haiti [3], Indonesia [4], Japan [5], and Nepal [6]—have reported that survivors continued to rely on emergency food during mid- (days to weeks) to long-term (weeks to months) periods, depending on the area and scale of the disaster [2]. For example, after the Great East Japan Earthquake on March 11th, 2011, distribution of fresh vegetables, meat, fish, and dairy products with well-balanced proteins and vitamins [5] was difficult [2,7,8,9]. Even one month later, survivors’ diets were largely limited to long-shelf life food, which had a high percentage of carbohydrates [5,7]. In some areas, disasters prior to the earthquake already had diminished rations, exacerbating the dearth of nutrients. To make matters worse, the earthquake damaged the main industrial nutrition supplement provider [10].

The scarcity of fresh fruits and vegetables resulted in deficiencies of corresponding nutrients such as dietary fiber and vitamin C. A survey of those in temporary shelters in Ishinomaki, Japan linked a lack of dietary fibers normally found in fresh fruits and vegetables, with an increased number of gastrointestinal symptoms [7]. These symptoms may have also contributed to decreased food intakes among 23% of the 236 survivors surveyed one month after the disaster [7]. Diets rich in carbohydrates likewise caused high blood glucose levels [7]. Research conducted 15 weeks after the Great East Japan Earthquake found that cardiovascular diseases had increased significantly [11]. Vitamin C is known to prevent cardiovascular diseases [12]. However, vitamin C was deficient at the time in previously healthy people [10]. The lack of proper nutrition over prolonged periods can thus result in various health issues [13]. Similar situations can occur in Tokyo, where rations consist of crackers, pregelatinized rice, instant noodles, and rice [2]. Although Japan is prone to large-scale disasters, one household survey revealed that most households did not store rations or did so insufficiently [2]. As access to nutrients during disaster situations has proven to be challenging, alternative nutrient sources must be explored and better understood.

In this study, we examine urban agriculture (UA) as one possible disaster preparation food source to supplement the emergency foods. Disaster preparation food [2] is defined as: “*food needed to maintain psychological and physical health for disaster survivors between the time a disaster occurs and when life returns to normal*” [2] (p. 46). According to this definition, disaster preparation food includes food with a short-shelf life that can be used until distribution of ingredients is restored [2]. Furthermore, an assessment of pre-earthquake investment after the 2010 earthquake in Haiti identified UA as a source of local food security and nutrition [14]. UA is broadly defined as the growing of plants and raising of animals inside the city [15,16], in the present study a focus is made on vegetable production. People engaged with UA have reported positive impacts on psychological health, nutrition [17,18,19], and community resilience [20]. Although more research is required, a systematic literature review showed positive associations with dietary diversity [21]. Therefore, we hypothesize that UA may very well be a viable disaster preparation food source if available in disaster struck areas.

Previous UA self-sufficiency studies addressed annual self-sufficiency of the total vegetables [t] [22,23,24,25]. However, disasters are intractable and impact at random [26]. Therefore, the present study aims to quantify UA vegetable production and the resulting nutritional self-sufficiency throughout the year for mitigating post-disaster situations. The nutritional self-sufficiency in this study is estimated according to the mean recommended intake by nutrient compared to the availability of that nutrient from agricultural production.

## 2. Materials and Methods

### 2.1. Study Design

**Figure 1:**
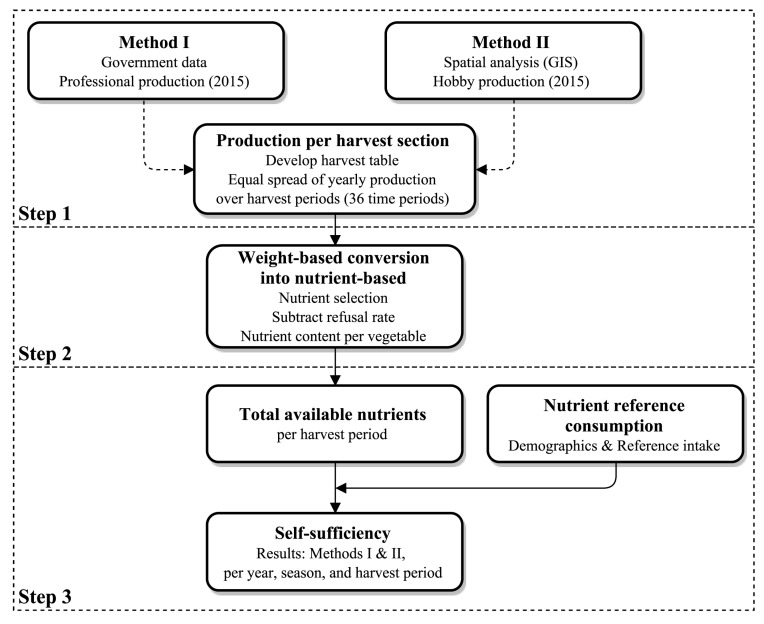
Flowchart of analysis.

The year-round production of vegetables [t] was estimated by assuming a constant level of production across vegetable harvest periods. Next, the weight of each harvest [t] was converted into nutrients [kg] and their total availability calculated. A reference consumption of each nutrient [kg] was estimated by multiplying dietary reference intakes with population statistics. The population statistics were categorized according to age and gender in correspondence to the nutrient reference intakes. In addition, the unit of each nutrient was converted from mg to kg because the original unit was intended for a per capita estimation and the present study estimates the entire population. Finally, self-sufficiency [%] throughout the year was quantified by dividing the available nutrients from UA with the reference consumption of the population, as detailed in Figure 1.

### 2.2. Step 1: Production of Vegetables Throughout the Year

Disasters can occur at any given time of year [26]. Therefore, as a disaster preparedness study, we felt that estimating the available harvest in anticipation of such occurrences at any given time of the year was a critical factor. In the selected case study, three types of full-soil UA were identified [22,27]: (1) Professional UA, which is similar to its rural counterparts but located within the city boundary; (2) Experience UA, land farmed by hobby users but owned by professionals. The professionals provide planting plans and practical advice to optimize the yields of the hobby farmers; and (3) Allotment UA, which are maintained by hobby farmers and often have a high variety in crops [22,27,28,29]. A Japanese municipal survey supplied data on the production of professional UA. However, data on the production of hobby UA was unavailable. Therefore, two methods were developed to estimate the total production of vegetables in the study site. Method I uses the aforementioned government survey data; Method II analyzes land use data from a Geographic Information System.

#### 2.2.1. Method I

Data from the 2015 Tokyo Metropolitan Agricultural Products Production Survey [30] was utilized. This survey on professional UA is conducted every 5 years by the Tokyo Metropolitan Government and lists the annual production and total area by vegetable type. The vegetables produced in the present case study were retrieved for use in steps two and three as shown in Figure 1.

#### 2.2.2. Method II

Information on allotment and experience UA [22,27] was collected and processed in a Geographic Information System (GIS) (ArcGIS ver. 10.3, Esri, Redlands, CA, USA). High-tech indoor and rooftop farms were excluded because they are susceptible to earthquakes. Through field observations and interviews with the UA section of the municipality, and with local professional and hobby farmers (2015–2016), it was found that both experience and allotment farms were present in the case study. Each type has their own visual characteristics. Because the plots located on experience farms are grown by hobby farmers under the strict guidance of a professional farmer, all the plots have similar crop patterns and choices of crops. Therefore, the fields are visually similar to each other. Allotment farms are also grown by hobby farmers; however, because some of these farmers have no knowledge on agricultural activities and they are not receiving guidance by a professional farmer, these plots have a more disordered appearance due to the high diversity of crop combinations in the different plots [22,27].

Hobby UA areas were identified according to the three steps developed by Sioen et al. (2016) as follows: (1) access to the municipality database [31,32] to retrieve the location of each hobby farm according to type (experience or allotment); (2) confirmation and documentation of their locations and a systematic scan of the entire ward with Google Earth Pro (ver. 7.1.8, , Google, Mountain View, CA, USA) to identify remaining undocumented farms; and (3) ground confirmation with Google Street View. The analysis was updated according to 2016 study site satellite imagery [22]. To reduce the margin of error of the identification through satellite imagery, typical examples of each type of the documented farms were randomly selected and visited on site for confirmation of the type and its location (Jul. 2016). An additional accuracy assessment was conducted with the help of land use data in GIS from the Tokyo Metropolitan Government [33], indicating the individual plot sizes of all land uses. The documented locations and sizes from the present study were fitted according to the plots from the land use map.

The total area of each type of farmland was multiplied with an average production indicator. This indicator shows the average production (vegetables [kg] in a square meter) from previously reported samples (for each type 5 plots were analyzed over a one year time frame) (Table 1).

The proportion of each vegetable in the total production of allotment and experience farms was also estimated based on the same samples [27]. This was important to determine the total nutrient content in each hobby UA area because nutrient contents vary by vegetable. Finally, the total production (*P*) of vegetable (*i*) from farm type (k) was estimated:

(1) $$P_{i}=x_{i}\overset{f}{\underset{k=1}{\sum }}A_{k}Y_{k}$$

where $x_{i}$ is the proportion of vegetable i [-]; $A_{k}$ is the area of farm type k = 1...f [m^2^]; and $Y_{k}$ is the yield of farm type k = 1…f [kg/m^2^].

**Figure 2:**
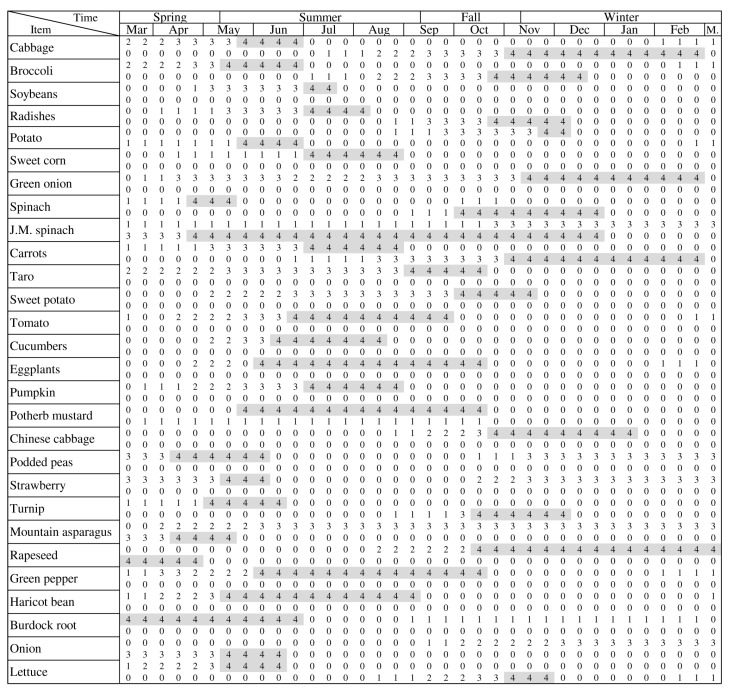
Planting and harvesting schedule by vegetable and season [34]. Each month was divided in three time periods (beginning, middle, and end). J.M. spinach: Japanese mustard spinach. Legend: 0 = No activity; 1 = Seeding; 2 = Planting; 3 = Growing; 4 = Harvesting.

For both Methods I and II, the production by vegetable was estimated throughout the year by equally distributing the annual production across the harvest periods. This means that each vegetable can be gradually harvested according to the need in time as long as it is within its harvesting period. In addition, the total production [t] was developed by aggregating the vegetable production derived from Methods I and II. To do so, a harvesting schedule was developed with data from literature [34] for vegetables grown in the study site. Place specific data is important because climatological and geographical conditions of the site and the type of vegetables grown can have an influence on the nutritional self-sufficiency. Some vegetables had multiple growing seasons. The schedules classified months in three time periods (beginning, middle, and end) according to the seeding, planting, growing, and harvesting periods, which we adopted in the present study. This resulted in 36 time periods for one year, each consisting of 10.14 days. The harvest periods are categorized according to the growing seasons as shown in Figure 2.

### 2.3. Step 2: Conversion of Vegetable Weights to Nutrients

The Ministry of Health, Labour and Welfare (MHLW) of Japan conducts an annual National Health and Nutrition Survey that lists the food sources from which dietary nutrients in Japanese diets are derived [35]. The 2015 survey lists 29 nutrients from several food sources. Nutrients for which fruits and vegetables contribute more than 20% of the total nutrient consumption in Japanese diets were analyzed in the present study. Furthermore, The Kagawa Nutrition University Publishing Division provides a list of nutrient content and average refuse rates of vegetables from common preparation and cooking methods in Japan. This data was retrieved from the 7th edition of the Tables of Food Composition (2016) [36]. The estimated refuse was subtracted, and multiplied by the remaining vegetable matter for each time period for each of the identified nutrients. This supplies us with the amounts by vegetable of each available nutrient in the respective time section. Finally, the nutrients provided by all vegetables at each time section were then totaled.

### 2.4. Step 3: Self-Sufficiency

Estimates of self-sufficiency rates were extrapolated from recommended dietary allowances and population statistics of the study area. Recommended dietary allowances were taken from a list of per capita daily dietary reference intakes for Japan developed by the Japanese MHLW. This list is updated every 5 years based on the latest scientific research [37]. The recommended dietary allowance was retrieved, or if unknown, the adequate intake of the selected nutrients per age group and gender for the year 2015 were obtained. Population statistics by age and gender were acquired from the portal site of Official Statistics of Japan developed by the Ministry of Internal Affairs and Communications (2015). The population was then categorized according age and gender specifications established by the Dietary Reference Intakes for Japanese (2015) [37] as shown in Table 2 [38]. Population statistics are available by one-year age groups. The first life year of both males and females was further divided into two groups for compatibility with dietary reference intakes. Children younger than 5 years old were excluded from the self-sufficiency calculation of dietary fibers because of the lack of scientific evidence on the reference intake for this group. Besides these factors, previous studies reported a higher nutrient demand for certain life stages (e.g., when pregnant or lactating) [39]. However, a low fertility rate of 1.24 was observed in the case study [40], which means that this target group is limited, and there was no data available for pregnant and lactating women in Nerima, which is crucial for the nutrient demand. Additionally, pre-disaster diseases that can cause variations in nutrients suggestions—the case of diabetes patients—were not considered because of limitations in the data availability. However, conducting the estimations with the available data as shown in Table 2 can still produce the results that are in line with the aim of the study. The total requirements for the remaining nutrients were calculated for each age category and gender by multiplying the reference intakes for Japan with the population.

Self-sufficiency over the course of the year was estimated by dividing the produced nutrition by the reference consumption of each time section. The following equation was developed for the estimation of self-sufficiency:

(2) $$\eta_{j,t}=\frac{\sum_{i=1}^{v}h_{i,t}P_{i}\left(1-r_{i}\right)N_{i,j}}{C_{j}}$$

where $h_{t}$ is the harvest rate per time section t [-]; $P_{i}$ is the production of vegetable i = 1…v [kg]; $r_{i}$ is the refuse rate of each vegetable [-]; $N_{i,j}$ is the nutrient content per vegetable [mg/kg]; and $C_{j}$ is the reference consumption of nutrient per time section [mg].

The resulting self-sufficiency rates by nutrient were compared with the self-sufficiency rate in the total vegetable weight [kg], which was estimated based on the targeted per capita recommendation of the mean daily intake of vegetables (350 g) set by the Japanese MHLW [41].

### 2.5. Setting

Nerima ward is second most populous of the 23 special wards (urban areas) of Tokyo’s prefecture, with 15,019 people/km² (2015) over a surface area of 48.08 km^2^. Despite Japan’s population decline, the population in the ward grew from 716,124 residents in 2010, to 721,709 residents in 2015 [38]. Some 222,650 residents make their living in the tertiary industry, but only 1180 professionals in the primary industry and 43,009 in the secondary industry [38]. Nerima ward has a long history with farming. For example, the family of Shiraishi Y. has been farming in the ward since Edo period (1603–1868) [42] and there are farmers that specialize in vegetable species primarily grown in the ward during that time period (e.g., Nerima Daikon [43], which is a type of radish) [44]. The ward is one of few that has its own UA section. Registered farmers can receive subsidies for their investments, a platform for knowledge exchange, and promotion during public events (e.g., farming historical species is supported by Nerima’s UA section) [44]. The ward was selected because of its substantial area of agricultural land in densely populated areas and its general contribution to UA in Tokyo [45].

Nerima’s large share of UA within Tokyo’s special wards is the result of its slower urban growth rate compared to other wards in the sixties [46]. Located on the edge of the upland plateau, Nerima’s soil is good for vegetable production [47], which motivated farmers to protect their farmlands. The government established the City Planning Act in 1968 [48] and designated open spaces (including farmlands) in Tokyo as Urbanization Promotion Areas (UPA). These areas were intended for development within the next 10 years, however, many places remained UA because the urban growth rate slowed down. UPA lands were taxed as urban land uses, which caused a substantial financial burden for remaining farmers. This was also the case for UA in Nerima.

**Figure 3:**
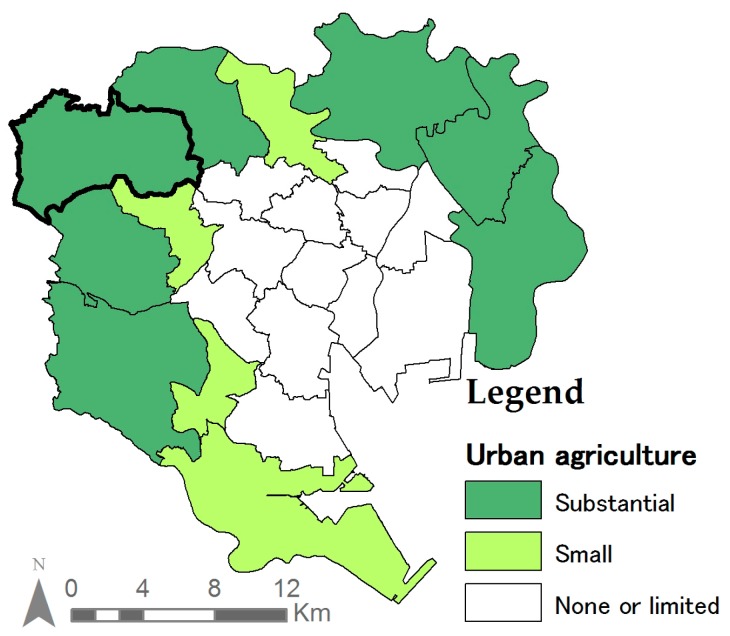
Tokyo’s 23 special wards where UA can be found (Nerima ward bolded) [33].

Under the City Planning Act, the Productive Green Land Act was enacted in 1974 and revised in 1992 [48]. The act reduced land taxations for farmers if a 30-year commitment to UA was made. It successfully enabled farmers to continue their activities by delaying development, simultaneously protecting UA. This resulted in the large presence of remaining agricultural land uses in the outer belt of Tokyo’s 23 wards (Figure 3). Despite Nerima’s important ranking in UA, the number of people engaged in professional agricultural activities has decreased from 1890 people in 1970 to 714 people in 2005, a decrease of more than 60% over a period of 25 years. However, the decline has stabilized at 642 people since 2010 [49]. Nerima ward today, contains the highest number of UA area (180.23 ha) in the 23 special wards of Tokyo [33].

### 2.6. Subjects

The Nerima ward residents (categorized according to gender) were the subjects of this study. 4814 people (0.67%) of unknown age [38] were excluded from the study because age is an important factor in estimating the reference consumption of nutrients. Life stages (e.g., pregnant, or lactating) and health conditions (diabetic), which can affect the reference consumption for those individuals were not considered in the present study because no data was available for the population. Despite these limitations, the estimation can provide a general outcome for the entire case study population. The numbers of subjects totaled 716,895 people.

## 3. Results

**Figure 4:**
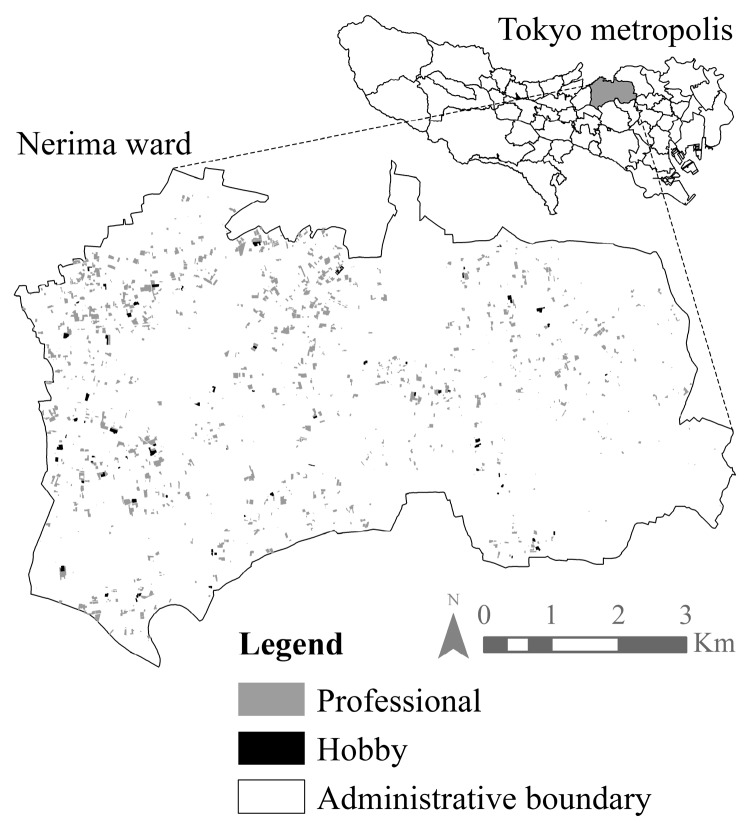
Urban agriculture in Nerima ward, Tokyo. Hobby UA consist of both allotment and experience UA. Basemap: Tokyo Metropolitan Government [22,31].

Figure 4 shows the spatial analysis (Step 1, Method II) that identifies 53 allotment UA plots (7.38 ha) and 26 experience UA plots (7.32 ha) in Nerima ward. The figure also shows the distribution of the 1396 professional UA farms (180.23 ha) used in Method I. The average size of an UA plot in the ward was determined as 1321.59 m^2^. The yields estimated by Methods I and II were 4776 tons and 884 tons, respectively, totaling to 5660 tons. Table 3 shows the yield of each vegetable obtained from governmental data by Method I. By omitting the refuse [36] and factoring in the number of harvest periods, the harvests ready for consumption in each time period were added further quantifications in Step 2. The same procedure was applied for estimating production from hobby UA, using data from the previous studies in Method II. The results are shown in Table 4.

Table 5 shows results regarding estimated weights and nutrients.

The weight-based estimations from the recommended per capita vegetable intake and production in Nerima ward indicate a self-sufficiency rate of 6.18%. This weight-based estimation also enables comparison with previous self-sufficiency studies. The nutrient-based results were estimated with the reference intake per nutrient. Nine nutrients met the selection criteria described in Step 2. The required intake of these nutrients for the entire population of Nerima was converted to kilograms (Table 5). The self-sufficiency rates estimated according to each Method in step 3, reflect the reference intake per nutrient. Nerima was most self-sufficient in vitamin K, vitamin C, folic acid, dietary fiber, and potassium, which are derived from vegetables. Lower levels of self-sufficiency were found for vitamin B6, vitamin A, vitamin E, and calcium also coming from vegetables. The average nutritional self-sufficiency rates according to Methods I and II were 2.48% and 0.38%, respectively. These combine to the aggregated average nutritional self-sufficiency rate of 2.86%. Although it had a small representation compared to that of professional UA, hobby UA still contributed to 0.98% of the ward’s vegetable self-sufficiency. Fluctuations were also found throughout the seasons based on utilized species and their resulting harvests as shown in Table 6.

**Figure 5:**
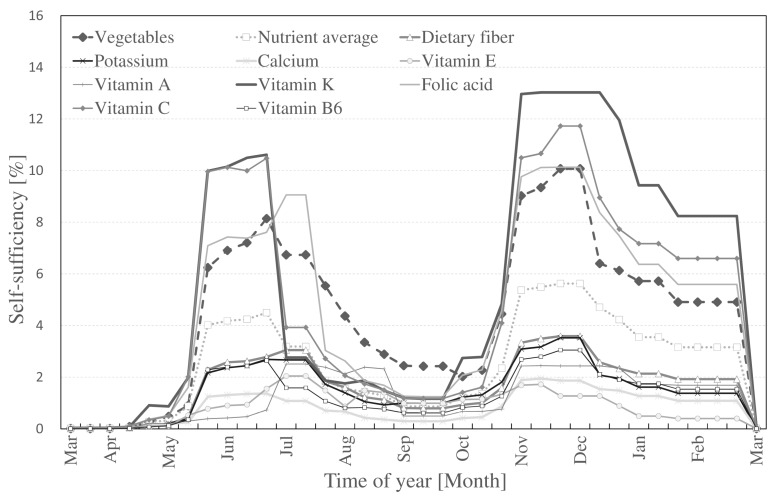
Self-sufficiency in Nerima ward from professional UA (Method I).

**Figure 6:**
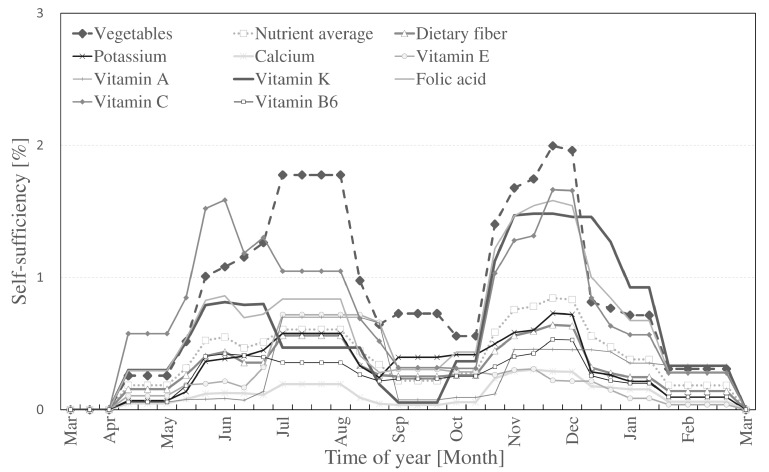
Self-sufficiency in Nerima ward from hobby UA (Method II).

The self-sufficiency rates for the nine selected nutrients and overall self-sufficiency of vegetables are shown in Figure 5 (Method I) and Figure 6 (Method II). The figures are based on the three time periods per month as shown in the harvest table produced in Step 1. Variations in self-sufficiency are present because of two main factors: (1) type of nutrient; (2) time of year. According to the nutrient scale from professional UA in Figure 4, vitamin C showed the highest self-sufficiency compared to other nutrients while vitamin E was found to have the lowest rates. In Figure 6, the highest self-sufficiency was also vitamin C, however, due to the variation in vegetables cultivated (as shown in Table 3 and Table 4), calcium was the lowest. Winter vegetables are harvested by the end of February, resulting in the lowest self-sufficiency in March and beginning of April. According to the harvest table, these times are mostly planting periods. The highest self-sufficiency was observed from July to the end of August (summer harvest) and from the end of October to the beginning of December (winter harvest). Although professional UA contributed more in sheer amount because of its larger presence, hobby UA contributed more with less land and experienced a more constant supply than professional farms.

**Figure 7:**
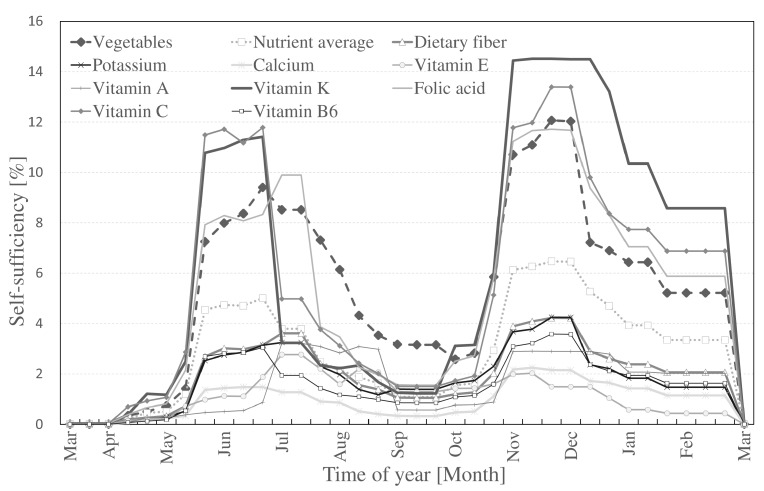
Aggregated self-sufficiency in Nerima ward from professional and hobby UA (Method I and II).

Finally, the combined vegetable and nutritional self-sufficiency rates from Methods I and II are shown in Figure 7. Variations throughout the year can be attributed to fluctuations in the planting and harvesting seasons (Figure 2). Vegetable weight-based self-sufficiency averaged 5.24% across the year, higher than the nutritional average of 2.86%. The hobby UA analyzed with Method II contributed to a more stable supply of UA vegetables and nutrients in Nerima ward. Averages in self-sufficiency were found for vitamin K (6.15%), followed by vitamin C (5.50%), folic acid (5.15%), dietary fiber (1.96%), and potassium (1.82%), vitamin A (1.54), vitamin B6 (1.54%), vitamin E (1.13), and calcium (0.96%).

## 4. Discussion

UA contributed on average 2.86% self-sufficiency of the nine nutrients selected in this case study. In other words, depending on the season and nutrient, about 20,503 people out of 716,895 people can be self-sufficient in nutrients from UA in the present case study. Step 1 of the analysis, estimated production from professional UA using governmental data (Method I) and production from hobby UA (allotment, experience farming) by conducting a spatial analysis (Method II). To understand the impact of this study, we discuss the results in four sections as follows: (1) comparison with other case studies and contextualization of the results; (2) impact and target of nutritional self-sufficiency for post-disaster situations; (3) the role of UA in disasters; and (4) limitations and future work.

### 4.1. Comparison with other Case Studies and Contextualization of the Results

We postulate that the high self-sufficiency rate in Nerima compared to previous weight-based studies [23] may very well be caused by the active engagement of the local government in promoting and subsidizing UA activities. Specifically, the local government subsidizes farmer’s investments in new facilities and provides a unique platform for knowledge exchange and quality improvement - as the municipality is one of the few in the country to have its own UA section. In addition, investments by innovative farmers in Nerima, creating viable businesses [48], led to the creation of experience farms. Shiraishi Y. [42] established this new type of farming, generating his income by providing agricultural experiences to urbanites rather than through professional agricultural production [22,32,42]. Experience UA proved to have higher yields compared to professional or allotment farming [27]. Further democratizing UA can thus increase the self-sufficiency in the case study area. The overall popularity of UA for education, leisure, and the self-cultivation of food can be attributed to a high number of public initiatives and events (e.g., annual radish harvesting rally or blue berry picking events [44]) [28]. These led Nerima to become the municipality in Tokyo prefecture with the largest share of hobby farms [22].

Though numerous UA studies have estimated vegetable self-sufficiency, we found none that estimated nutritional self-sufficiency. For example, Grewal and Grewal [23] conducted a study in Cleveland (OH, USA) and found 1.7% self-sufficiency for fresh produce, despite its lower population density in the city (2241 people/km^2^ [50]) compared to Nerima (15,019 people/km^2^ [38]). The result drew from annual yield and consumption of UA produce. For comparison purposes, we applied the same weight-based analysis to the present case study and discovered a 6.18% annual vegetable self-sufficiency. We also found that the weight-based self-sufficiency varied throughout the year (average after subtraction of the refuse: 5.24%). These results are higher than previous findings because of high yields and urban planning policies protecting agricultural activities in Japanese cities [51].

Prior studies failed to estimate vegetable production in different times of the year [22,23]. Because disasters can affect communities at any time [26], knowledge of nutrition availability from UA at any given point of year will lead to improved disaster preparedness and speedier emergency food provision. We have thus estimated the contribution of UA to the nutritional self-sufficiency throughout the year. The present study corroborates that vegetable yields fluctuated during the year, effecting the corresponding self-sufficiency rates. The highest average self-sufficiency rates from combining Methods I and II were in winter (4.20%), followed by summer (3.08%), and fall (2.40%). Little to no self-sufficiency was discovered in spring (0.21%). Indeed, nutrient self-sufficiency at the time of the March 11th Great East Japan Earthquake in the present case study was a critically low as 0.02%, putting public health at risk. At that time, the rations in the Tohoku area (northeast Japan) were already low from typhoons, flooding’s, and eruptions of active volcanoes that occurred before the earthquake. Furthermore, damage of the main supplement provider exacerbated the nutrition crisis at the time [10]. These results confirm the vulnerability of the area during times of low self-sufficiency and potentially increased resilience during times of higher self-sufficiency.

### 4.2. Impact and Target of Nutritional Self-Sufficiency for Post-Disaster Situations

The present case study shows the potential value of UA as a source of disaster preparedness food. When considering the total population, the mean nutritional self-sufficiency was found to be 2.86%. However, this result has a bigger impact when targeting evacuees of a disaster. A simulation in Tokyo conducted by the Tokyo Metropolitan Government (TMG) [52] predicted that a magnitude 7.3 earthquake under Tokyo Bay North Area (worst case scenario) would force 3,390,000 residents to evacuate and be in need of food and shelter by the next day. This is 26% of the city’s population that requires food. If this ratio of potential evacuees in the first phase after a disaster [2] (up to three days depending on the scale and location) is applied to the present study area, the mean nutritional self-sufficiency would be 11%. This means that 20,503 evacuees can have immediate access to sufficient nutrients from within the disaster struck area. As this research has indicated, self-sufficiency levels vary by nutrient and time of year. Nutrient self-sufficiency levels range from 23.65% in vitamin K to 3.67% in calcium. Seasonally, the highest mean nutritional self-sufficiency according to this estimation would be in winter (16.50%) and the lowest in spring (0.97%). However, survivor’s primary need during this first phase after the disaster is in carbohydrates, which is already provided by conventional emergency food [2].

Nutritional self-sufficiency becomes crucial during the mid-term phase (days−months) after a disaster to avoid gastrointestinal symptoms and cardiovascular diseases due to dependence on carbohydrate based emergency foods [1]. Greater numbers of evacuee’s move from the affected area during this phase. Previous studies highlighted higher nutritional needs of vulnerable populations (e.g., young children, elderly, patients, pregnant, and lactating women [5]), which is a fraction of the 26% target ratio set in the estimation above. Due to the lack of data on life stages, health conditions, and migration patterns of the population after a disaster, we estimated this phase for the nutritional self-sufficiency of children (0–14 years old) and the elderly (50+ years old) based on the simulation by TMG for the mid-term phase after a disaster. The mean nutritional self-sufficiency for 96,442 people in Nerima was found to be 22.71% (21,902 people), about double that of the previous phase, and still underestimated due to the described limitations in data. The highest from the selected nutrients according to this estimation would be 33.34% in winter and the lowest 1.64% in spring. Again, the self-sufficiency rate was highest for vitamin K (48.50%) and lowest in calcium (7.31%), but with vitamin K exceeding the necessary level for half of the evacuees 16 out of 36 time periods seen in Figure 2.

**Figure 8:**
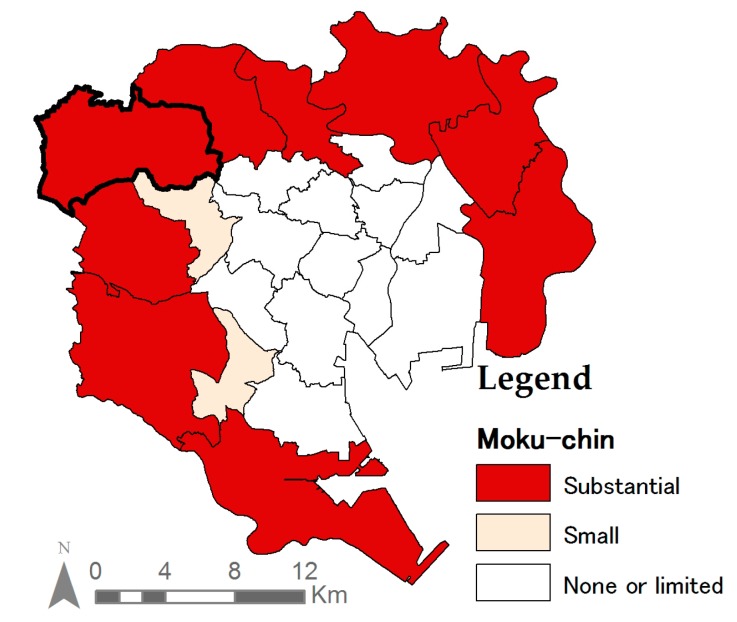
Vulnerable wards with high-densities of Moku-chin (characterized by narrow roads, causing greater risks in emergency situations [53]) in the 23 special wards of Tokyo (Nerima ward bolded) [33].

UA can play a more important role depending on the area of the disaster. During the expansion of Tokyo in the sixties, a large number of wooden rental houses (Moku-chin) was built in areas surrounding the city to meet the increasing housing demand [46,53]. In these areas, basic infrastructure was often disregarded due to a failure to adopt planning concepts [46]. These areas are characterized by narrow roads with limited connectivity, vulnerable to disasters [53], and difficult to provision [46]. In contrast, the inner core of the city was planned with infrastructure and wider roads. Thus, emergency food can be distributed from rations provided by large corporations. Therefore, UA has a greater importance as disaster preparation food in the wards characterized with Moku-chin (Figure 8). Figure 3 and Figure 8 together indicate that UA could indeed function as a potential food source in these vulnerable areas.

UA can play a further role in long-term recovery processes (months−years) [2]. The conventional distribution system would be recovering during this phase. The primary role of UA during this phase shifts from a source of nutrients to a source of survivors’ self-esteem and psychological health [17].

### 4.3. Role of Urban Agriculture in Disasters

The present study confirms the valuable role of UA [54] to complement emergency food provisions in disaster preparedness as a local source of food with crucial nutrients [2]. UA can complement survivor diets with nutrients beyond those in provided rations and external emergency foods which are rich in carbohydrates [7]. This diversified diet with vegetables mainly providing dietary fiber can help prevent several health issues previously reported in post-disaster health studies around the world [3,4,5,6]. Studies link higher intakes of dietary fiber with numerous health benefits [55]: the prevention of gastrointestinal symptoms, higher blood glucose levels, and higher blood pressure [7]. Additionally, a dearth of vegetable consumption can lead to vitamin deficiencies [10], and non-specific complaints [56], such as colds and coughs [5]. Overall, there is a consensus that vegetables are beneficial for health [13]. Though evidence is limited, some reports mentioned the positive role of UA in post-disaster situations [14] and indicated that UA may very well have significant positive health outcomes during post-disaster situations.

Large disasters cause a variety of mental health disorders in disaster survivors [57]. The World Health Organization reported that UA can play such a role in its relief: “*Supporting self-reliance is important to enhance the capacities and self-esteem of the affected population and may contribute to reducing dependence on food aid*” [1] (p. 34). The day-to-day benefits of physical exercise and social interactions with UA practices can therefore lead to better health during recovery periods. A study conducted in the United Kingdom revealed a significant correlation between consumption of healthy foods and perceived stress and depression [58]. Although further research on post-disaster situations is needed, food from UA can help alleviate such mental disorders.

UA can improve disaster awareness, as engaged populations can increase disaster preparedness. An increase in the proliferation of hobby UA can result from this engagement and lead to more diverse food sources that meet the local needs and preferences [19]. The strengthened presence of UA can also reduce the hurdle of food distribution processes in heavily affected areas with large populations. It was reported after the Great East Japan Earthquake that smaller emergency shelters might have had improved nutritional conditions. Nutrition conditions were best where evacuees were few in number and gas supply was quickly restored [9]. In the case of densely populated megacities, complex utility systems and narrow roads [59], such as those in the present study area, will undoubtedly cause the distribution of meals to be even more challenging. Tis is in comparison to areas with relatively lower population densities such as those affected in northeastern Japan in 2011. Therefore, local distribution and consumption of fresh produce from UA can complement meals among a larger number of beneficiaries and increase the nutrient intake across heavily affected areas.

**Figure 9:**
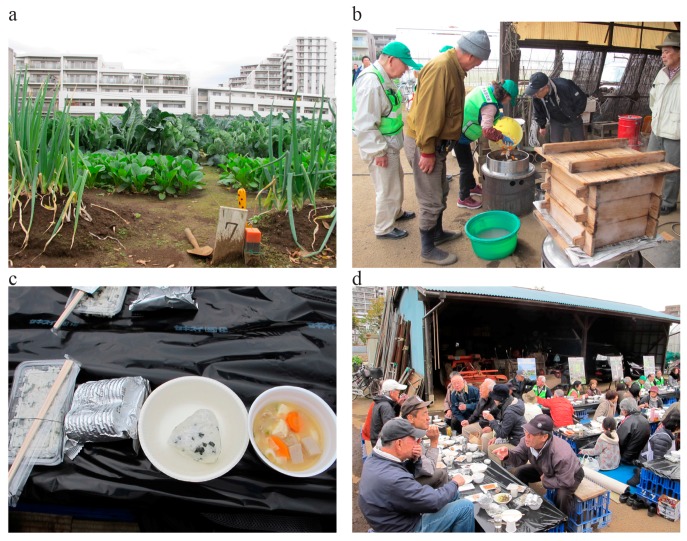
Disaster drill held in Nerima ward. (**a**) Urban farmland with a high diversity in crops; (**b**) Farmer and volunteers preparing soup with fresh vegetables from the farm in a portable gas stove; (**c**) Rice and crackers provided by the municipality as emergency food with freshly made soup containing vegetables from the farm; (**d**) People from the neighborhood familiarizing with each other and the farmer (photographs by the authors, November 2016).

The Nerima ward government has been exploring UA benefits for local provisioning by improving disaster awareness, and neighborhood familiarity with farmers for evacuation and sourcing food in emergencies. The local government took the initiative to engage professional and hobby farmers in a yearly disaster drill as an additional to help neighboring residents in disaster preparedness (Figure 9). During the drill, residents from the neighborhood familiarize themselves with the UA areas. Post-disaster studies have reported damages to utilities (electricity, gas, and water), which proved problematic for cooking [10]. Vegetables grown in Nerima ward, such as cabbages and carrots, can be consumed raw in emergencies. However, for certain nutrients, steaming or cooking the vegetables minimizes the nutritional loss compared to other cooking methods [60]. Cooking can also increase dietary fiber content per gram in some vegetables when heating removes excess water from the vegetables [13]. Local governments (including Nerima) have already prepared portable gas cookers for when utilities are unavailable [9]. Thus, this can lead to improving nutritional intake in times of disasters.

### 4.4. Limitations and Future Work

Our study has several limitations. Firstly, the nutrient content in some vegetables varies according to the harvest season. However, because such details were unavailable for all vegetables [36], only average contents were used in the current study. Secondly, the production from professional UA in Method I came from market-based government statistics that exclude direct sales and self-consumption [30]. Therefore, the actual production and corresponding self-sufficiency from Method I could be higher in reality. In contrast, the production from hobby UA in Method II may be overestimated because few studies have weighed the vegetable production from hobby UA [27]. Specifically, the indicators used for the estimations were drawn from five samples of allotment and experience farms. These samples were pioneering cases at the time. Thirdly, potential damage from disasters to farmlands needs to be considered in future studies. Previous earthquakes in Japan damaged farm equipment and buildings, but reported damage to vegetable fields was limited. Fourthly, previous post-disaster studies reported that pregnant and lactating women were groups in specific need for sufficient nutrients [39]. Also, survivors diagnosed with health issues before a disaster (e.g., diabetes patients) experienced higher risks compared to their healthy counter-parts. Due to data limitations, these factors were not incorporated in the present study. Fifthly, the nutritional self-sufficiency was estimated with the dietary reference intake for daily purposes [37]. However, the nutrient requirement during the first and second phase after a disaster could be reduced to the minimum in an emergency situation. Because of the focus on carbohydrates in emergency studies [1], there was insufficient data available to incorporate the minimum requirements of nutrients for the population in the present study. Despite these limitations, the study is able to estimate the nutritional contributions of UA for the general public. Lastly, we assumed that all agricultural lands in use are safe from heavy metals and other contaminants, and that the use of chemicals is kept within the government guidelines. Despite the above-mentioned limitations, the authors believe that the present study contributes to the advancement of self-sufficiency studies for post-disaster situations.

It is argued that self-sufficiency stands in contradiction to trade agreements [61], a response to the demand by people for fresh and safe food [62]. However, this research shows that food self-sufficiency can increase the resilience of communities. Therefore, local food systems may seem redundant in the face of international trade but are invaluable for disaster preparedness. Future studies should identify a target self-sufficiency rate for each nutrient by simulating different scales of disasters, while maintaining trade agreements. In addition, they should explore the contributions of UA in different areas of the city according to their land use patterns (ratio of urban and agricultural land uses) as well as potential contributions from other lands that are currently unutilized (e.g., vacant land [33]).

## 5. Conclusions

UA has potential to supplement rations and other emergency foods as disaster preparedness food, depending on the time of year. In the present case study, the mean self-sufficiency rates varied according to season (winter (4.20%), summer (3.08%), fall (2.40%), and spring (0.21%)). If contemporary emergency food rations are to prevent diet-caused symptoms among survivors, such rations should be better strategized with local UA availability and the time of year the disaster takes place. The present case study showed variations in the mean self-sufficiency by nutrient (vitamin K (6.15%), vitamin C (5.50%), folic acid (5.15%), dietary fiber (1.96%), potassium (1.82%), vitamin A (1.54), vitamin B6 (1.54%), vitamin E (1.13), and calcium (0.96%)) indicating the importance to address self-sufficiency studies on scale of nutrients. Also, it was discussed that focusing on the vulnerable target groups (age groups 0–14, and 50+) of refugees, the mean self-sufficiency of selected nutrients in the present case study was 22.71%. This study has implications for policies and emergency response strategies around the world to increase the intake and availability of vegetables with crucial nutrients provided by UA during post-disaster situations.

The two main findings of this study were: (1) UA can provide a valuable contribution to the nutrient provisioning of survivors during different stages after a large disaster; (2) emergency food must be targeted according to the time of year the disaster takes place to meet the needs of survivors.

## Figures and Tables

**Table 1:** Production by farmland type [22,27].

No.	Land Use Type	Indicator [kg/m^2^]
1	Allotment	4.16
2	Experience	6.91

**Table 2:** Population by age and gender distribution in 2015 [38].

Age Group	Total	Male	Female
0–5 months *	2935	1497	1438
6–11 months *	2935	1497	1438
1–2 years	11,543	5906	5637
3–5 years	16,930	8720	8210
6–7 years	11,381	5888	5493
8–9 years	11,046	5732	5314
10–11 years	11,221	5717	5504
12–14 years	18,115	9370	8745
15–17 years	18,642	9559	9083
18–29 years	101,874	49,695	52,179
30–49 years	225,450	113,735	111,715
50–69 years	168,816	84,328	84,488
70+ years	116,008	47,393	68,615
Unknown **	4814	2572	2242
Total	721,709	349,037	367,858

* Equally divided based on data 0–11 months population dataset, ** Not utilized in the present study.

**Table 3:** Harvest per vegetable and net weight per time period according to Method I.

No.	Vegetable Item	Harvest [t] [30]	Refuse [%] [36]	Harvest [periods]	Harvest per Period [t]
1	Cabbage	1973.00	15%	16	104.82
2	Radishes	557.00	10%	9	55.7
3	Tomato	307.00	3%	10	29.78
4	Eggplant	255.00	10%	14	16.39
5	Carrot	217.00	3%	18	11.69
6	Potato	211.00	10%	6	31.65
7	Chinese cabbage	198.00	6%	9	20.68
8	Broccoli	153.00	50%	11	6.95
9	Green onion	147.00	40%	11	8.02
10	Japanese mustard spinach	119.00	15%	25	4.05
11	Soybeans	111.00	45%	2	30.53
12	Spinach	86.00	10%	12	6.45
13	Cucumbers	84.00	2%	7	11.76
14	Sweet corn	76.00	50%	6	6.33
15	Turnip	73.00	9%	11	6.04
16	Sweet potato	72.00	9%	5	13.10
17	Taro	61.00	15%	5	10.37
18	Pumpkin	24.00	10%	6	3.60
19	Strawberry	10.00	2%	3	3.27
20	Green pepper	10.00	15%	14	0.61
21	Potherb mustard	8.00	15%	15	0.45
22	Podded peas	6.00	9%	6	0.91
23	Mountain asparagus	6.00	35%	4	0.98
24	Haricot beans	6.00	3%	12	0.49
25	Rapeseed	3.00	0%	20	0.15
26	Burdock root	3.00	10%	18	0.15
Total		4776.00			384.91

**Table 4:** Harvest per vegetable and net weight per time period according to Method II.

No.	Vegetable Item	Harvest [t]	Refuse [%] [36]	Harvest [periods]	Harvest per Period [t]
1	Radishes	203.39	10%	9	20.34
2	Chinese cabbage	99.20	6%	9	10.36
3	Tomato	83.88	3%	10	8.14
4	Cabbage	69.08	15%	16	3.67
5	Cucumbers	60.08	2%	7	8.41
6	Potato	58.96	10%	6	8.84
7	Taro	50.08	15%	5	8.51
8	Carrots	44.95	3%	18	2.42
9	Podded peas	43.13	9%	6	6.54
10	Green onion	31.85	40%	11	1.74
11	Pumpkin	26.74	10%	6	4.01
12	Broccoli	26.64	50%	11	1.21
13	Eggplants	20.90	10%	14	1.34
14	Onion	19.14	6%	4	4.50
15	Spinach	17.43	10%	12	1.31
16	Sweet potato	13.63	9%	5	2.48
17	Green pepper	8.33	15%	14	0.51
18	Lettuce	6.28	2%	7	0.88
Total		883.70			95.21

**Table 5:** Annual required nutrients, available nutrients, and self-sufficiency rates.

Name	Required [kg]	Available [kg]		Self-Sufficiency [%]		
		Method: I	II	I	II	I & II
Vegetables in weight	91,583,336.00	4,776,000.00	883,701.00	5.21	0.96	6.18
Dietary fiber	4,552,058.45	75,493.58	13,801.21	1.66	0.30	1.96
Potassium	686,243.03	10,338.25	2160.88	1.51	0.31	1.82
Calcium	171,473.72	1441.21	196.97	0.84	0.11	0.96
Vitamin C	24,565.73	1174.72	176.10	4.78	0.72	5.50
Vitamin E	1566.47	13.73	3.98	0.88	0.25	1.13
Vitamin B6	321.67	4.20	0.77	1.30	0.24	1.54
Vitamin A	190.11	2.39	0.53	1.26	0.28	1.54
Folic acid	58.34	2.65	0.36	4.54	0.61	5.15
Vitamin K	37.03	2.06	0.22	5.57	0.58	6.15
Nutrient average				2.48	0.38	2.86

**Table 6:** Average self-sufficiency by method and season.

Name	Method I [%]				Method II [%]			
	Spring	Summer	Fall	Winter	Spring	Summer	Fall	Winter
Vegetables in weight	0.11	2.60	2.01	3.78	0.09	0.48	0.39	0.42
Dietary fiber	0.06	1.95	1.40	2.29	0.08	0.41	0.34	0.29
Potassium	0.07	1.79	1.57	1.91	0.03	0.42	0.45	0.28
Calcium	0.06	0.84	0.70	1.30	0.02	0.13	0.12	0.15
Vitamin E	0.05	1.25	1.21	0.75	0.05	0.47	0.29	0.12
Vitamin A	0.07	1.42	0.92	1.86	0.03	0.39	0.15	0.36
Vitamin K	0.33	4.74	4.27	9.66	0.15	0.53	0.57	0.86
Folic acid	0.18	4.98	3.53	6.78	0.15	0.67	0.69	0.75
Vitamin C	0.17	4.97	3.33	7.63	0.29	1.01	0.60	0.70
Vitamin B6	0.04	1.45	1.15	1.88	0.03	0.33	0.28	0.23
Nutrient average	0.11	2.60	2.01	3.78	0.09	0.48	0.39	0.42
